# The first complete mitochondrial genome sequences of *Neopsylla specialis dechingensis* and *Neopsylla stevensi sichuanyunnana*, with an assessment of their phylogenetic placement

**DOI:** 10.3389/fvets.2026.1865746

**Published:** 2026-06-23

**Authors:** Lanling Tian, Jun Wu, Tianguang Ren, Mingna Duan, Shaobo Tang, Rui Hou, Wei Gu, Dandan Jiang, Xing Yang

**Affiliations:** 1 Integrated Laboratory of Pathogenic Biology, College of Preclinical Medicine, Dali University, Dali, Yunnan, China; 2 Department of Ophthalmology, Dali Bai Autonomous Prefecture People’s Hospital, Dali, Yunnan, China; 3 School of Government Administration, Baoshan University, Baoshan, Yunnan, China; 4 Department of Infection, The First Affiliated Hospital of Dali University, Dali, China; 5 The Key Laboratory of Infectious Diseases of Yunnan Provincial Education Department, Dali, China; 6 The Clinical Medical Sub-Center of Infectious Diseases of Yunnan Province, Dali, China; 7 School of Public Health, Dali University, Dali, Yunnan, China

**Keywords:** gene structure, mitochondrial genome, *Neopsylla specialis dechingensis*, *Neopsylla stevensi sichuanyunnana*, phylogentic relationship

## Abstract

Fleas are common blood-sucking external parasites found on mammals; they serve as important vectors for various pathogenic microorganisms and pose a serious threat to human and animal health. Mitochondrial genomes have been widely used in studies such as DNA barcoding for species identification and phylogenetics. Therefore, in this study, we performed sequencing of *Neopsylla specialis dechingensis* and *Neopsylla stevensi sichuanyunnana* on the Illumina NovaSeq platform. Genome assembly and gene annotation were subsequently done to obtain their complete mitochondrial genomes. Besides the two newly sequenced specimens, sequence information of 35 extra flea taxa downloaded from the NCBI database was adopted for overall comparative evaluation. The results showed that a significant AT base bias was present in the mitochondrial sequences of all 37 flea species tested. The Ka/Ks values calculated for the 13 PCGs were all lower than 1, which indicates that these genes are subject to persistent purifying selection. The Pi values indicated that ATP8 exhibited the highest variability, while ND5 was the most conserved. Phylogenetic trees were built based on 13 PCGs of 37 species, with *Boreus elegans* assigned as the outgroup, and both trees share highly identical topological structures. Both *N. s. dechingensis* and *N. s. sichuanyunnana* were clustered within the family Ctenophthalmidae. Furthermore, both trees supported the monophyly of the family Pulicidae with auto-expanded support values and posterior probabilities of 100 and 1, respectively. As important disease vectors, fleas pose a substantial risk to both human and veterinary health. However, complete mitochondrial genome data for fleas are currently extremely limited. For this reason, ongoing expansion of the flea mitochondrial genome database is critical to advancing related research efforts.

## 1 Introduction

Fleas are classified under phylum Arthropoda, class Insecta, and order Siphonaptera; they are external parasites of warm-blooded animals (1). The two species studied in this research are: *N. s. dechingensis*, collected from *Eothenomys miletus* in Zhenxiong, Yunnan; and *N. s. sichuanyunnana*, collected from *Niviventer confucianus* in Tengchong, Yunnan. *N. s. dechingensis* is one of the species with the widest ecological niche among small mammalian fleas in the Tibetan region of northwestern Yunnan, while *N. specialis* is an important vector flea in plague-endemic areas (2); The sampling site for *N. s. sichuanyunnana*, Tengchong, Yunnan, is a natural reservoir for plague, and this subspecies is endemic to China. Fleas are not host-specific and can parasitize a variety of hosts, thereby facilitating the cross-species transmission of diseases (3). Rodents are the primary hosts of fleas; only about 6% of fleas parasitize birds, and they are found all over the world (4). Fleas undergo four developmental stages—egg, larva, pupa, and adult—before reaching maturity, making them insects that undergo complete metamorphosis (5). The adult’s body can be broadly divided into three parts: the head, thorax, and abdomen. It is small in size, with a smooth, tough exoskeleton. Its color ranges from yellowish-brown to dark brown. The body is laterally flattened, wingless, and has three pairs of legs, making it an excellent jumper (6). Fleas not only bite and suck blood, causing direct harm to humans and animals or leading to allergic dermatitis and anemia, but they may also cause rashes during the feeding process, or even result in suppurative reactions at the bite site (7, 8). Fleas can also transmit *Rickettsia typhi* and *Bartonella henselae* through their feces, or *Yersinia pestis* through contaminated mouthparts. Various zoonotic pathogens, such as *Dipylidium caninum* and *Francisella tularensis*, have also been found in fleas. Globally, the economic losses caused by fleas amount to $15 billion annually (9–13). Fleas meet all the criteria for being the primary vectors of plague and serve as an important indicator for monitoring the early stages of a plague outbreak (14). According to historical records, the plague has caused three major global outbreaks, resulting in massive deaths among both humans and animals (15). As of December 2024, more than 3,000 species or subspecies across 19 families have been reported worldwide (16). However, the GenBank database currently contains complete mitochondrial genome sequences for only about 20 species of fleas, which is extremely limited and hinders the prevention and management of flea-transmitted diseases (2).

The maintenance of mitochondrial function hinges on SIRT3-mediated homeostatic regulation. As the primary mitochondrial deacetylase, SIRT3 activates metabolic substrates to sustain oxidative phosphorylation and ATP production, while also regulating mitochondrial dynamics (fission and fusion) to preserve network integrity (17). In fields such as comparative genomics, evolutionary biology, phylogenetic analysis, and population genetics, the mitochondrial genome has been widely utilized as a molecular structure with a high density of genetic information (18, 19). Mitochondria are double-membraned organelles that primarily supply energy to organisms and serve as the site of cellular aerobic respiration; they contain DNA and possess their own genetic material and specific genetic information (20, 21). Compared to nuclear DNA, mitochondrial DNA analysis is more sensitive and has a higher likelihood of successful PCR amplification. However, mitochondria do not function entirely independently; most of their physiological processes are regulated by nuclear DNA, and they must work in concert with nuclear genes in a semi-autonomous manner to support the organism’s normal activities (22, 23). Studies have shown that the phylogenetic information contained in a complete mitochondrial genome far exceeds that of individual genes, making it a valuable tool for phylogenetic and evolutionary studies across various taxonomic levels (24–26). For the first time, we sequenced the complete mitochondrial genomes of *N. s. dechingensis* and *N. s. sichuanyunnana* and performed a phylogenetic analysis. This work addresses a critical gap in the mitochondrial genetic data for these two flea species, expands available resources for taxonomic and evolutionary studies on fleas, and establishes a data foundation to support future prevention and management of flea-transmitted diseases.

## 2 Materials and methods

### 2.1 Specimen collection and morphological identification

*N.s.dechingensis* and *N.s.sichuanyunnana* were collected from Zhenxiong County, Zhaotong City (27°37′N, 104°37′E), Yunnan Province, China from the body of *E.miletus* and from the body of *N.confucianus* in Tengchong City (24°38′N–25°52′N, 98°05′E–98°46′E), Yunnan Province, China. The study used 31 individuals of *N. s. dechingensis* and 27 individuals of *N. s. sichuanyunnana.* The specimens were preserved in 95% ethanol for subsequent morphological identification and DNA extraction. After mounting, the specimens were photographed and morphologically identified according to Fauna Sinica: Insecta, Siphonaptera (27). Genomic DNA was extracted using the DNeasy Blood and Tissue Extraction Kit from QIAGEN (Hilden, Germany) following the manufacturer’s protocol.

### 2.2 Mitochondrial sequencing, assembly, and annotation

In this study, whole-genome shotgun (WGS) sequencing was used to construct multiple sequencing libraries with different insert sizes. Paired-end (PE) sequencing was performed using next-generation sequencing (NGS) technology on the Illumina NovaSeq high-throughput sequencing platform. After cleaning and filtering the raw sequencing data, quality control was performed using the fastp software^1^ to obtain high-quality, clean sequences. Subsequently, SPAdes v3.15.4 (28) and GetOrganelle v1.7.7.0^2^ to perform *de novo* assembly of the sequences. The resulting contigs were then aligned against the NCBI nt database using the blastn tool to identify mitochondrial-related sequences. Finally, Bandage v0.8.1^3^ was used to visualize the assembly relationships of the mitochondrial fragments. The assembled sequences were corrected using Pilon v1.18 (29) to obtain complete and reliable mitochondrial genome sequences. The assembled complete mitochondrial genome sequences were uploaded to the MITOS web server^4^ for functional annotation (30).

### 2.3 Characteristics of mitochondrial genome sequences

Here, we performed a comparative genomic analysis focusing on the mitogenomes of two newly sequenced flea species alongside 35 additional taxa retrieved from the NCBI database; detailed information on these species is provided in Table 1. The nucleotide composition of individual genes was quantified using DNA Star v11.1 software; base skew was calculated using the formulas AT-skew = (A − T)/(A + T) and GC-skew = (G − C)/(G + C) (31). The base skew data is plotted using Origin v2025. Use DNASP 6.0 (32) to calculate the synonymous substitution rate (Ka) and the non-synonymous substitution rate (Ks), and analyze nucleotide diversity (Pi) using a sliding window (window size = 100 bp, step size = 25 bp). Using CodonW v1.4.2 software, the relative synonymous codon usage (RSCU) of 13 protein-coding genes was quantified and evaluated, followed by graphical representation using R to systematically analyze their codon usage patterns.

**Table 1:** Basic information on the 37 flea species analyzed.

Family	Species	Accession no.	Length (bp)			
			Complete genome	PCGs	tRNAs	rRNAs
Ceratophyllidae	*Amphalius spirataenius*	OR855715	14,825	11,135	1,435	2078
Ceratophyllidae	*Ceratophyllus anisus*	NC_073017	15,875	11,137	1,433	1997
Ceratophyllidae	*Ceratophyllus wui*	NC_040301	18,081	11,057	1,432	2019
Ceratophyllidae	*Citellophilus tesquorum dzetysuensis*	PV693698	16,458	11,138	1,429	2075
Ceratophyllidae	*Citellophilus tesquorum mongolicus*	PV693696	15,373	11,042	1,431	2076
Ceratophyllidae	*Citellophilus tesquorum sungaris*	PP418872	15,345	11,030	1,429	2029
Ceratophyllidae	*Jellisonia amadoi*	NC_022710	17,031	11,119	1,431	2080
Ceratophyllidae	*Macrostylophora euteles*	OR774969	16,027	11,114	1,431	2067
Ceratophyllidae	*Nosopsyllus laeviceps*	PP838812	16,533	11,143	1,433	1970
Ctenophthalmidae	*Ctenophthalmus quadratus*	NC_072692	15,938	11,126	1,405	2033
Ctenophthalmidae	*Ctenophthalmus yunnanus*	NC_085277	15,801	11,118	1,415	2031
Ctenophthalmidae	*Neopsylla hongyangensis*	PP133648	15,832	11,144	1,419	2087
Ctenophthalmidae	*Neopsylla specialis*	NC_073019	16,820	11,142	1,408	2053
Ctenophthalmidae	*Stenischia humilis*	NC_073020	15,617	11,118	1,424	2051
Ctenophthalmidae	*Stenischia montanis yunlongensis*	OR780663	15,651	11,118	1,425	2065
Ctenophthalmidae	*Stenischia montanis*	PP990561	15,889	11,124	1,425	2065
Ctenophthalmidae	*Stenoponia polyspina*	OR834393	14,933	11,124	1,405	2081
Ctenophthalmidae	*Neopsylla specialis dechingensis*	PX854823	17,530	11,139	1,413	2054
Ctenophthalmidae	*Neopsylla stevensi sichuanyunnana*	PX833418	15,357	11,145	1,417	2056
Hystrichopsyllidae	*Hystrichopsylla weida qinlingensis*	NC_042380	17,173	11,129	1,424	2010
Ischnopsyllidae	*Thaumapsylla breviceps orientalis*	PP973737	15,631	11,147	1,434	2083
Leptopsyllidae	*Amphipsylla qinghaiensis*	PQ571081	15,579	11,135	1,429	2082
Leptopsyllidae	*Frontopsylla diqingensis*	PP083946	16,153	11,125	1,437	2093
Leptopsyllidae	*Frontopsylla spadix*	NC_073018	15,085	11,144	1,439	2067
Leptopsyllidae	*Leptopsylla segnis*	NC_072691	15,785	11,138	1,420	2054
Leptopsyllidae	*Paradoxopsyllus custodis*	OQ627398	15,375	11,111	1,432	2070
Pulicidae	*Ctenocephalides canis*	NC_063710	15,609	11,082	1,412	2098
Pulicidae	*Ctenocephalides felis felis*	MK941844	15,418	11,084	1,420	2031
Pulicidae	*Ctenocephalides felis felis*	MW420044	20,911	11,094	1,415	2087
Pulicidae	*Ctenocephalides felis*	NC_049858	20,873	11,093	1,416	2086
Pulicidae	*Ctenocephalides orientis*	NC_073009	22,189	11,082	1,425	2,102
Pulicidae	*Pulex irritans*	NC_063709	20,337	11,095	1,439	2087
Pulicidae	*Xenopsylla cheopis*	MW310242	18,902	11,064	1,426	2,109
Stivaliidae	*Aviostivalius klossi bispiniformis*	OR774970	16,593	11,059	1,436	2,108
Stivaliidae	*Aviostivalius klossi bispiniformis*	PP963728	18,669	11,050	1,434	2,107
Tungidae	*Tunga penetrans*	PV426769	17,279	11,094	1,426	2063
Vermipsyllidae	*Dorcadia ioffi*	NC_036066	16,785	11,134	1,436	2084

### 2.4 Analysis of phylogenetic positioning

The phylogenetic analysis was conducted using mitochondrial gene sequences from two newly sequenced species and 35 flea species from NCBI as the internal group, with *B. elegans* (accession number: HQ696579) serving as the outgroup, and then using the 13 PCGs from these 37 species. Phylogenetic trees were inferred via both maximum likelihood (ML) and Bayesian inference (BI) approaches. Sequences from these 38 species were extracted using Phylosuite v2 (33) software, followed by alignment with MAFFT v7.149 (34). The aligned sequences were then trimmed using gblocks v0.91b (35), and a maximum likelihood (ML) tree was constructed using IQ-TREE v1.6.12 (36) with 1,000 bootstrap repetitions. For Bayesian inference (BI), MrBayes v3.2.7 (37) was used with the GTR + F + I + G4 model identified by ModelFinder v2.2.0 (38) as the optimal model. The analysis ran for 1 million generations, with sampling performed every generation. The first 25% of tree samples were discarded as warm-up data, and only the subsequent stable samples were used to construct the phylogenetic tree, ensuring that the ASDSF value was less than 0.01. The final results were visualized in ITOL v7.5 (39).

## 3 Results

### 3.1 Morphological characteristics

**Figure 1:**
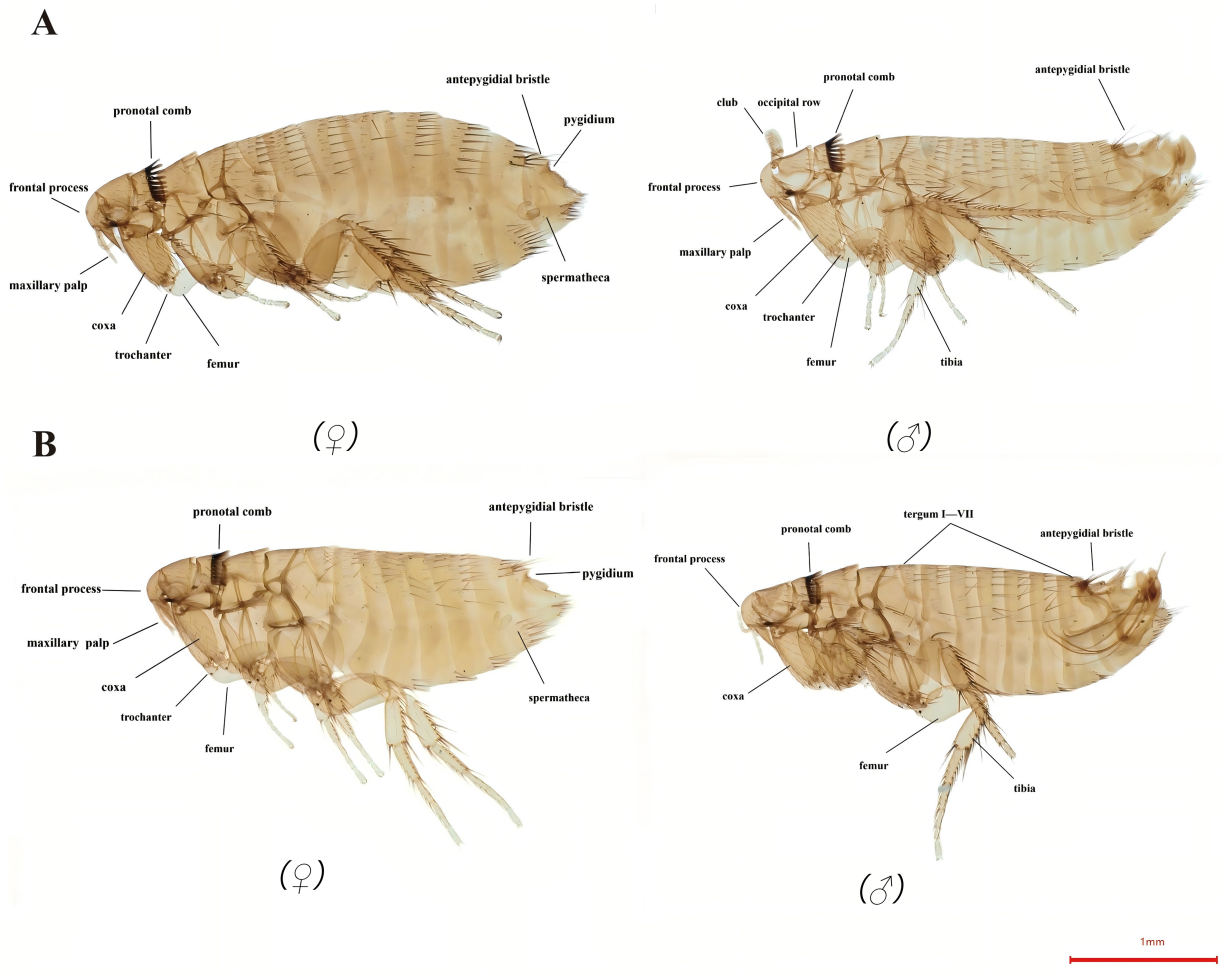
Illustrations of the morphological characteristics of *N. s. dechingensis* **(A)** and *N. s. sichuanyunnana* **(B)**.

Adult fleas are generally 1 to 3 mm long, with the largest reaching about 7 mm. Their body surface is covered with appendages such as bristles, spines, and hairs. The body is primarily partitioned into three sections: the head, thorax, and abdomen. The head is divided into the pre-antennal area and the post-antennal area by the antennal fossa. The mouthparts consist of a pair of maxillary palps, usually comprising four segments, and a pair of labial palps, typically comprising four to five segments. The thorax consists of the prothorax, mesothorax, and metathorax, with each segment bearing a pair of legs. The coxa of each leg is broad and flattened, particularly well-developed; the trochanter is short and narrow; the femur bears bristles of varying sizes and densities; and the tibia often has well-developed bristles. The abdomen consists of 10 segments: the first seven are pregenital segments, each composed of a dorsal and ventral plate, while the tenth segment is the anal segment. In *N. s. dechingensis*, the movable process is relatively broad, with its widest point located below the midpoint (♂); on the 7th abdominal plate, the lamella is often indented or notched, and its junction with the ventral lobe is typically sharply concave (♀). See Figure 1A; In *N. s. sichuanyunnana*, the movable process is narrower, longer, and straighter (♂); the frontal process is located below the midpoint of the frontal margin and is higher in males than in females; there is a single row of 6 to 7 frontal setae, and four ocular setae. See Figure 1B.

### 3.2 Comprehensive analysis of genomic features

**Figure 2:**
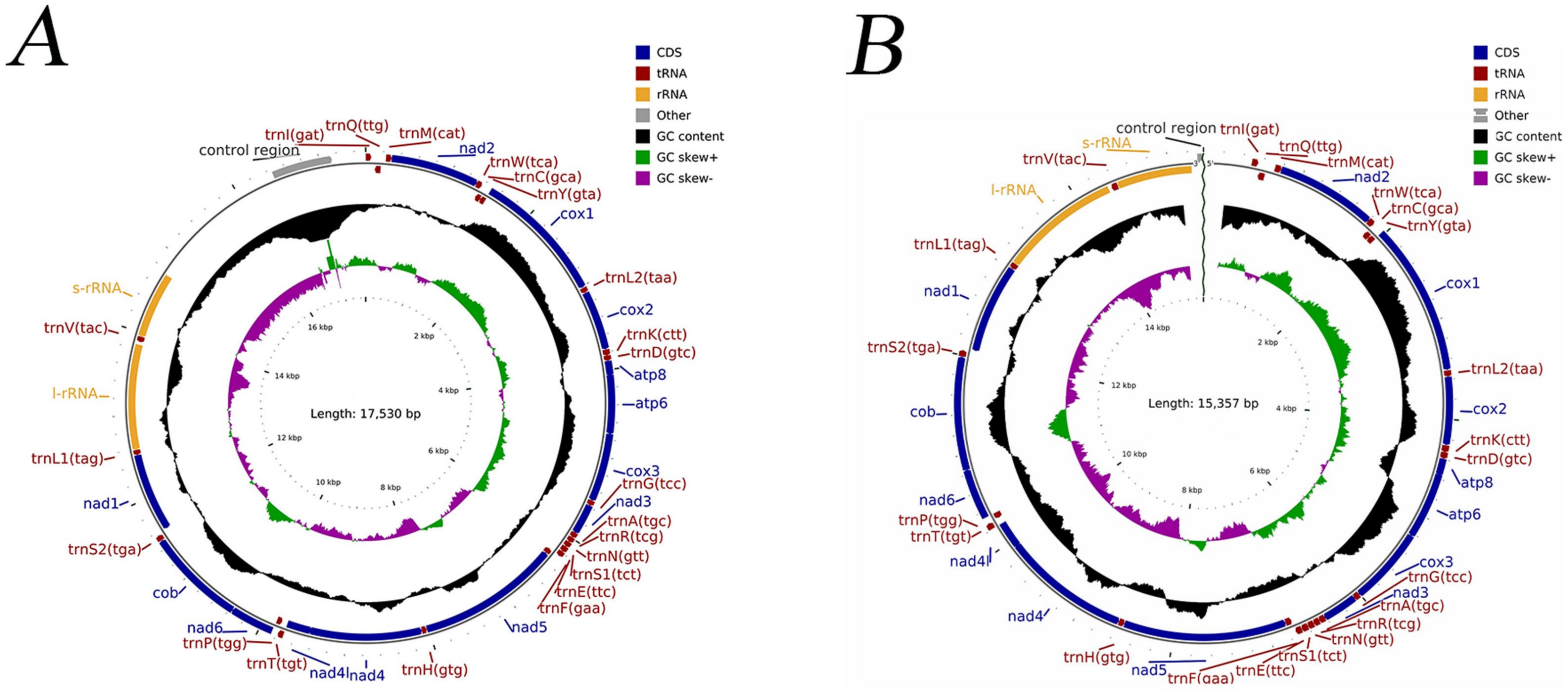
Circular maps of the complete mitochondrial genomes of *N. s. dechingensis* **(A)** and *N. s. sichuanyunnana* **(B)**.

**Figure 3:**
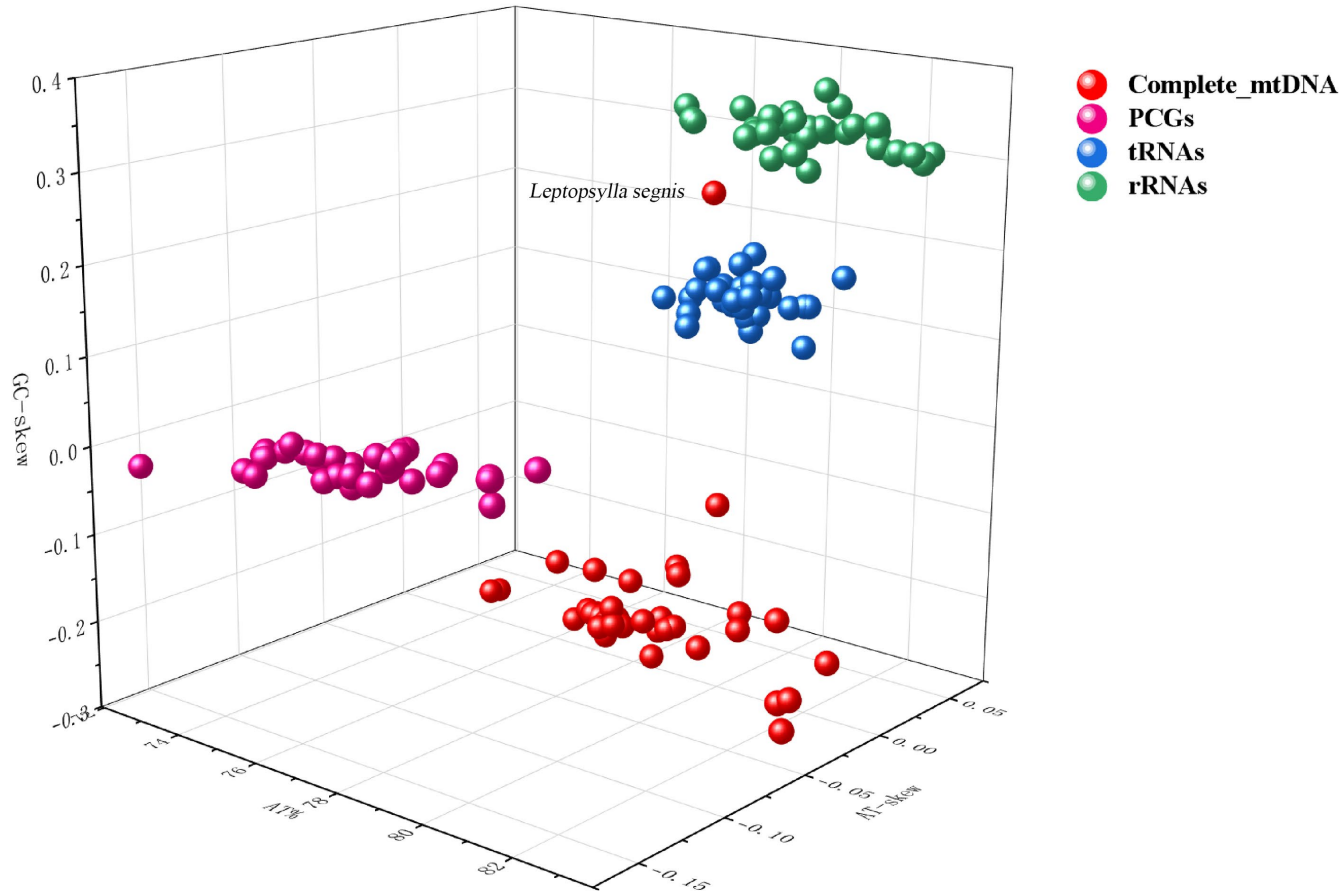
3D scatter plots (AT%, AT-skew, GC-skew) for 37 flea species.

The complete mitochondrial sequences ofthe two newly characterized species have been deposited in NCBI; the accession number for *N. s. dechingensis* is PX854823, and that for *N. s. sichuanyunnana* is PX833418. The genomes of both species contain 22 tRNAs, 13 PCGs, and two rRNAs. Both *N. s. dechingensis* and *N. s. sichuanyunnana* possess heavy chains containing 14 tRNAs and 9 PCGs, while the light chains contain 8 tRNAs, 4 PCGs, and two rRNAs. The former has 10 overlapping regions between genes, and the latter has 13 overlapping regions; the complete genomic structure is shown in Figure 2 where (Figure 2A shows *N. s. dechingensis*, and Figure 2B shows *N. s. sichuanyunnana*). Among the 37 species studied, the shortest mitochondrial genome sequence was 14,825 bp (*Amphalius spirataenius*), and the longest was 22,189 bp (*Ctenocephalides orientis*). This study generated a three-dimensional scatter plot by calculating the base composition of the 37 species, as shown in Figure 3. In the complete genome base composition of the 37 species, the AT% values ranged from 74.62 to 83.21%, demonstrating significant AT bias. AT skew values spanned from −0.0511 to 0.0236, while GC skew values ranged from −0.2678 to 0.2475, with the majority of estimates falling below zero, as shown in Table 2.

**Table 2:** A + T content, AT-skew and GC-skew of the complete mitochondrial genomes and functional regions (PCGs, tRNAs, rRNAs) from 37 flea species.

Species	Accession no.	Complete genome			PCGs			tRNAs			rRNAs		
		A + T (%)	AT-skew	GC-skew	A + T (%)	AT-skew	GC-skew	A + T (%)	AT-skew	GC-skew	A + T (%)	AT-skew	GC-skew
*Amphalius spirataenius*	OR855715	78.7	−0.0336	−0.1986	77.87	−0.1487	0.0430	79.65	0.0271	0.1370	81.33	0.0095	0.3351
*Ceratophyllus anisus*	NC_073017	78.54	−0.0221	−0.2314	76.39	−0.1464	0.0160	80.04	0.0166	0.1469	80.97	0.0142	0.3368
*Ceratophyllus wui*	NC_040301	76.71	−0.0166	−0.1836	76.87	−0.1473	0.0090	79.61	0.0211	0.1370	80.88	0.0116	0.3472
*Citellophilus tesquorum dzetysuensis*	PV693698	78.45	−0.0310	−0.2200	75.79	−0.1481	0.0304	79.78	0.0193	0.1419	81.69	0.0301	0.3368
*Citellophilus tesquorum mongolicus*	PV693696	78.25	−0.0293	−0.2177	76.63	−0.1438	0.0229	79.32	0.0185	0.1486	81.65	0.0289	0.3333
*Citellophilus tesquorum sungaris*	PP418872	78.07	−0.0292	−0.2172	76.42	−0.1484	0.0265	79.78	0.0193	0.1419	81.07	0.0334	0.3281
*Jellisonia amadoi*	NC_022710	79.17	−0.0202	−0.2596	76.71	−0.1511	0.0039	79.66	0.0211	0.1478	81.06	0.0095	0.3573
*Macrostylophora euteles*	OR774969	77.59	−0.0080	−0.2678	74.91	−0.1434	0.0100	80.36	0.0191	0.1459	80.31	0	0.3612
*Nosopsyllus laeviceps*	PP838812	78.10	−0.0287	−0.1654	78.17	−0.1431	0.0440	80.60	−0.0009	0.1583	81.47	0.0093	0.2986
*Ctenophthalmus quadratus*	NC_072692	79.45	−0.0135	−0.2256	77.75	−0.1397	0.0291	80.28	0.0142	0.1264	81.21	−0.0236	0.3455
*Ctenophthalmus yunnanus*	NC_085277	79.36	−0.0158	−0.2278	77.65	−0.1356	0.0197	80.07	0.0185	0.1206	81.49	−0.0187	0.3511
*Neopsylla hongyangensis*	PP133648	74.62	−0.0017	−0.2578	72.25	−0.1562	−0.0304	78.08	0.0144	0.1254	79.54	−0.0133	0.3630
*Neopsylla specialis*	NC_073019	77.27	0.0002	−0.2509	74.84	−0.1534	−0.0061	78.84	0.0108	0.1342	79.88	−0.0183	0.3511
*Stenischia humilis*	NC_073020	78.00	−0.0110	−0.2384	75.90	−0.1430	0.0198	79.71	0.0185	0.1765	81.33	−0.0120	0.3629
*Stenischia montanis yunlongensis*	OR780663	77.29	−0.0117	−0.2375	74.99	−0.1462	0.0054	79.09	0.0133	0.1678	81.07	−0.0143	0.3453
*Stenischia montanis*	PP990561	77.54	−0.0125	−0.2373	75.02	−0.1458	0.0061	79.09	0.0151	0.1678	81.07	−0.0143	0.3453
*Stenoponia polyspina*	OR834393	78.81	−0.0045	−0.2314	77.80	−0.1283	0.0016	79.64	0.0295	0.1818	81.84	−0.0112	0.3280
*Neopsylla specialis dechingensis*	PX854823	78.27	0.0085	−0.2654	75.15	−0.1542	−0.0072	78.98	0.0108	0.1448	79.84	−0.0183	0.3527
*Neopsylla stevensi sichuanyunnana*	PX833418	78.09	−0.0158	−0.2360	76.59	−0.1394	0.0134	79.18	0	0.1254	80.84	−0.0072	0.3452
*Hystrichopsylla weida qinlingensis*	NC_042380	80.59	−0.0297	−0.2208	78.01	−0.1557	0.0192	80.20	0.0123	0.1631	81.54	0.0055	0.3369
*Thaumapsylla breviceps orientalis*	PP973737	78.53	−0.0367	−0.2209	76.80	−0.1382	0.0139	79.50	0.0140	0.1361	81.23	0.0366	0.3555
*Amphipsylla qinghaiensis*	PQ571081	79.18	−0.0263	−0.2128	77.34	−0.1503	0.0163	80.13	0.0026	0.1479	81.51	0.0077	0.3455
*Frontopsylla diqingensis*	PP083946	75.46	−0.0275	−0.2281	75.23	−0.1397	0.0116	79.47	−0.0088	0.1186	81.18	0.0288	0.3756
*Frontopsylla spadix*	NC_073018	78.83	−0.0362	−0.2143	77.47	−0.1336	0.0127	80.19	0.0121	0.1088	82.39	0.0264	0.3407
*Leptopsylla segnis*	NC_072691	78.89	0.0236	0.2475	77.08	−0.1441	−0.0027	79.58	0.0071	0.1517	81.69	0.0107	0.3457
*Paradoxopsyllus custodis*	OQ627398	76.79	−0.0076	−0.2592	74.82	−0.1430	0.0043	80.31	0.0157	0.1489	80.34	−0.0006	0.3612
*Ctenocephalides canis*	NC_063710	78.52	−0.0170	−0.1816	79.25	−0.1472	0.0396	80.59	0.0264	0.1314	82.84	0.0253	0.3222
*Ctenocephalides felis felis*	MK941844	81.04	−0.0168	−0.2038	80.18	−0.1423	0.0442	80.85	0.0314	0.1324	82.77	0.0256	0.3200
*Ctenocephalides felis felis*	MW420044	82.88	−0.0443	−0.2374	80.19	−0.1423	0.0419	80.78	0.0306	0.1324	83.28	0.0219	0.3238
*Ctenocephalides felis*	NC_049858	83.13	−0.0444	−0.2298	80.20	−0.1431	0.0428	80.72	0.0324	0.0842	83.37	0.0259	0.3141
*Ctenocephalides orientis*	NC_073009	83.21	−0.0511	−0.2576	80.02	−0.1373	0.0099	81.12	0.0467	0.1599	83.16	0.0389	0.3164
*Pulex irritans*	NC_063709	80.02	−0.0271	−0.1462	78.07	−0.1464	0.0390	79.78	0.0122	0.1340	82.51	0.0232	0.3260
*Xenopsylla cheopis*	MW310242	82.83	−0.0105	−0.2212	80.31	−0.1195	0.0386	80.79	0.0087	0.1752	83.07	0.0034	0.3613
*Aviostivalius klossi bispiniformis*	OR774970	79.04	−0.0008	−0.1696	77.2	−0.1174	0.0218	80.15	0.0078	0.1368	81.36	0.0029	0.3537
*Aviostivalius klossi bispiniformis*	PP963728	79.74	0.0056	−0.0923	77.18	−0.1194	0.0214	80.13	0.0113	0.1228	80.97	0.0059	0.3466
*Tunga penetrans*	PV426769	82.01	−0.0198	−0.1766	79.13	−0.1414	0.0428	79.94	0.0228	0.1399	81.87	0.0266	0.3422
*Dorcadia ioffi*	NC_036066	80.71	−0.0064	−0.1977	78.10	−0.1419	0.0418	79.39	0.0263	0.1351	81.38	−0.0118	0.3196

### 3.3 Protein-coding gene analysis

**Figure 4:**
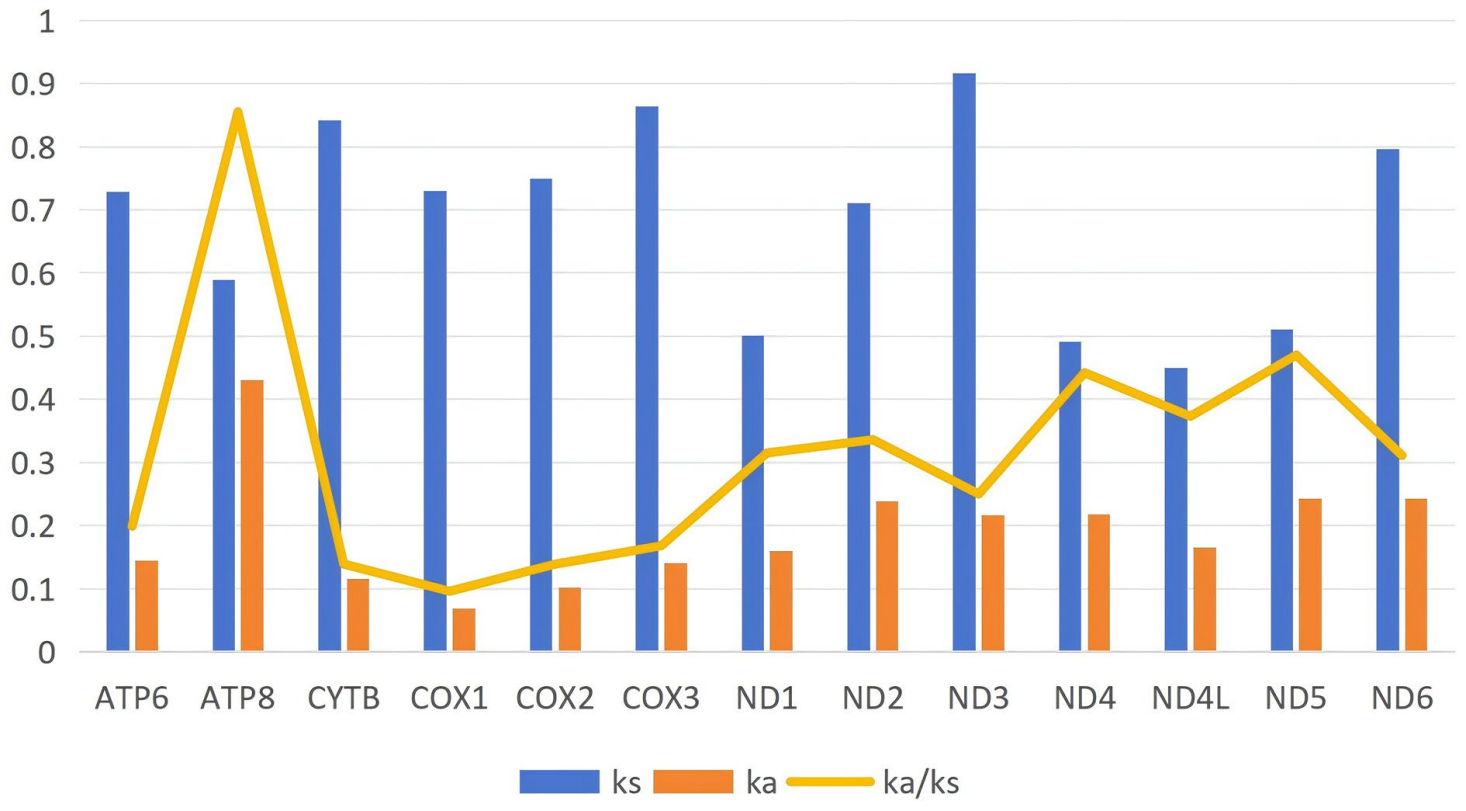
Ka/Ks plots for the 37 analyzed flea species.

**Figure 5:**
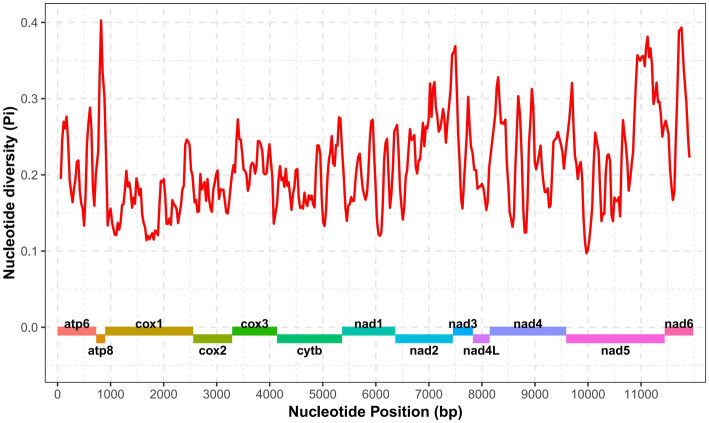
A map of nucleotide polymorphisms (Pi) for the 37 flea species analyzed in the study.

**Figure 6:**
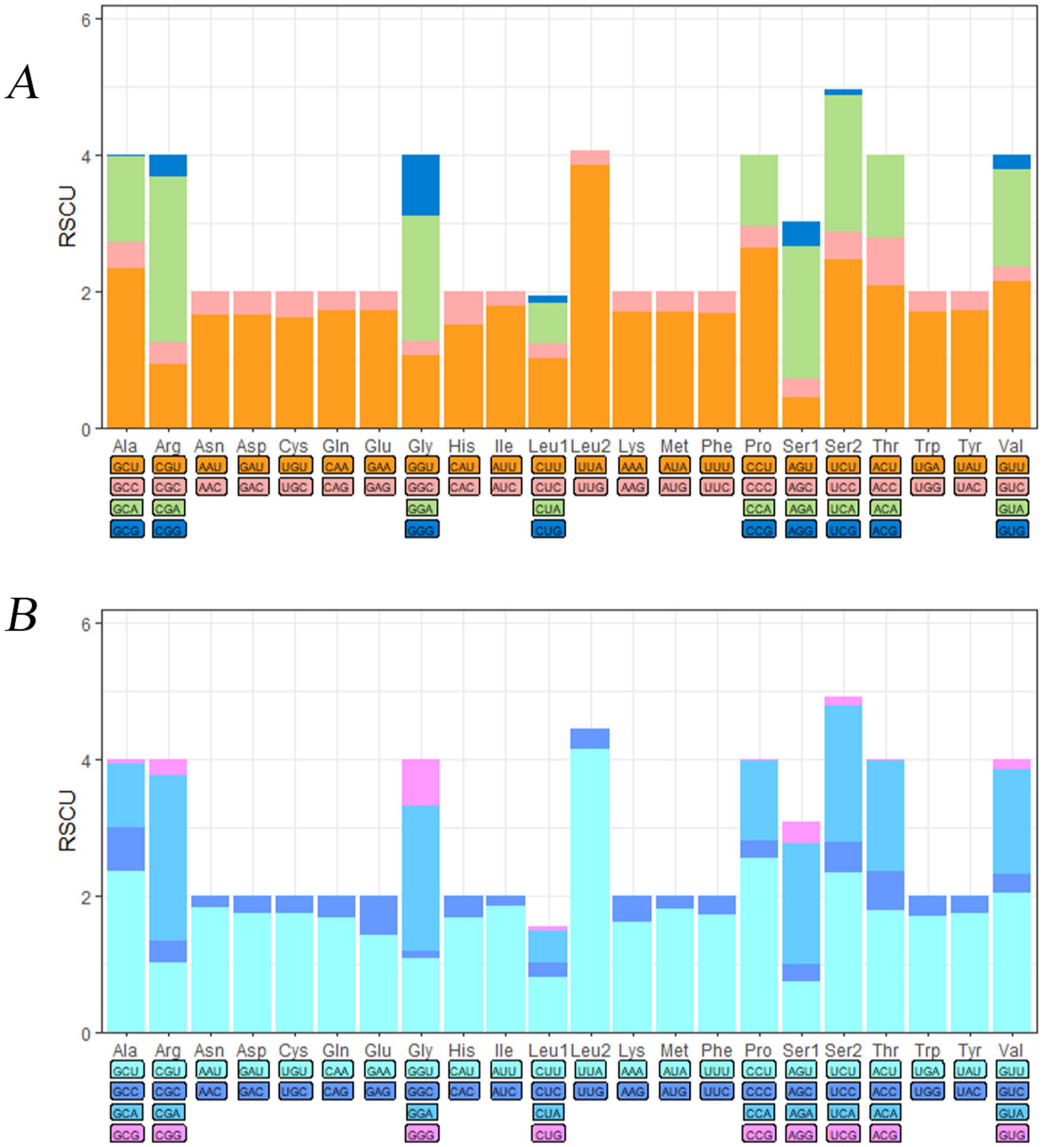
Relative synonymous codon usage (RSCU) plots for *N. s. dechingensis* **(A)** and *N. s. sichuanyunnana* **(B)**.

The AT% of PCGs across all 37 species ranged from 72.25 to 80.31%, with all AT skewness values being negative, ranging from −0.1562 to −0.1174. GC skewness was predominantly positive, with only a few negative values, ranging from −0.0304 to 0.0442, as shown in Figure 3 and Table 2. Analysis of evolutionary rates and nucleotide diversity (*π*) revealed that all ka/ks ratios were less than 1, indicating that these genes were under purifying selection. ATP8 exhibited the fastest evolutionary rate, while COX1 exhibited the slowest, as shown in Figure 4. Pi values spanned a range of 0.097 to 0.403, where ATP8 displayed the highest variability and ND5 the most conservative, as shown in Figure 5. The start codons of the 13 PCGs in *N.s. dechingensis* and *N.s. sichuanyunnana* all follow the ATN format, with the exception of the ND4 gene in *N.s. sichuanyunnana*, which uses the non-standard start codon GTG. Most stop codons follow the TAA or TAG format, while a small number use the incomplete T or TA format, as shown in Supplementary Table S1. Analysis of RSCU results shows that *N. s. dechingensis* encodes a total of 3,713 codons, while *N. s. sichuanyunnana* encodes 3,715 codons. In the former, leucine (Leu) and isoleucine (Ile) had the highest usage rates, accounting for 16.13 and 10.18%, respectively, while cysteine (Cys) and arginine (Arg) were used least frequently, accounting for 1.13 and 1.37%, respectively. Similarly, in *N. s. sichuanyunnana*, leucine (Leu) and isoleucine (Ile) were the most commonly utilized amino acids, making up 15.37 and 10.49% of the total, respectively, while cysteine (Cys) and arginine (Arg) were used least frequently, accounting for 1.07 and 1.37%, respectively. There are 28 RSCU values greater than 1 in *N. s. dechingensis* and 27 in *N. s. sichuanyunnana*, indicating a relatively high usage frequency. The codon that appeared most often in both species was UUA, while the least frequently used were CCG and ACG, with a frequency of 0 in *N. s. dechingensis* and 1 in *N. s. sichuanyunnana.* See Figure 6: *N. s. dechingensis* (Figure 6A) and *N. s. sichuanyunnana* (Figure 6B) and Table 3.

**Table 3:** (A) The codon usage profile for *N. s. dechingensis*, and (B) the codon usage profile for *N. s. sichuanyunnana*.

Codon	Count	RSCU	Codon	Count	RSCU	Codon	Count	RSCU	Codon	Count	RSCU
(A)											
UUU (F)	296	1.67	UCU (S)	99	2.46	UAU (Y)	168	1.73	UGU (C)	34	1.62
UUC (F)	58	0.33	UCC (S)	17	0.42	UAC (Y)	26	0.27	UGC (C)	8	0.38
UUA (L)	384	3.85	UCA (S)	80	1.99	UAA (*)	59	1.46	UGA (W)	75	1.69
UUG (L)	22	0.22	UCG (S)	4	0.10	UAG (*)	22	0.54	UGG (W)	14	0.31
CUU (L)	101	1.01	CCU (P)	76	2.64	CAU (H)	55	1.51	CGU (R)	12	0.94
CUC (L)	22	0.22	CCC (P)	9	0.31	CAC (H)	18	0.49	CGC (R)	4	0.31
CUA (L)	60	0.60	CCA (P)	30	1.04	CAA (Q)	58	1.71	CGA (R)	31	2.43
CUG (L)	10	0.10	CCG (P)	0	0.00	CAG (Q)	10	0.29	CGG (R)	4	0.31
AUU (I)	336	1.78	ACU (T)	73	2.09	AAU (N)	159	1.66	AGU (S)	18	0.45
AUC (I)	42	0.22	ACC (T)	24	0.69	AAC (N)	33	0.34	AGC (S)	11	0.27
AUA (M)	225	1.69	ACA (T)	43	1.23	AAA (K)	88	1.69	AGA (S)	78	1.94
AUG (M)	42	0.31	ACG (T)	0	0.00	AAG (K)	16	0.31	AGG (S)	15	0.37
GUU (V)	89	2.14	GCU (A)	71	2.33	GAU (D)	61	1.65	GGU (G)	53	1.06
GUC (V)	9	0.22	GCC (A)	12	0.39	GAC (D)	13	0.35	GGC (G)	11	0.22
GUA (V)	59	1.42	GCA (A)	38	1.25	GAA (E)	70	1.71	GGA (G)	91	1.82
GUG (V)	9	0.22	GCG (A)	1	0.03	GAG (E)	12	0.29	GGG (G)	45	0.90
(B)											
UUU (F)	294	1.73	UCU (S)	91	2.33	UAU (Y)	187	1.75	UGU (C)	35	1.75
UUC (F)	46	0.27	UCC (S)	18	0.46	UAC (Y)	27	0.25	UGC (C)	5	0.25
UUA (L)	396	4.16	UCA (S)	78	2.00	UAA (*)	64	1.51	UGA (W)	79	1.70
UUG (L)	28	0.29	UCG (S)	5	0.13	UAG (*)	21	0.49	UGG (W)	14	0.30
CUU (L)	77	0.81	CCU (P)	73	2.56	CAU (H)	61	1.67	CGU (R)	13	1.02
CUC (L)	19	0.20	CCC (P)	7	0.25	CAC (H)	12	0.33	CGC (R)	4	0.31
CUA (L)	46	0.48	CCA (P)	33	1.16	CAA (Q)	64	1.68	CGA (R)	31	2.43
CUG (L)	5	0.05	CCG (P)	1	0.04	CAG (Q)	12	0.32	CGG (R)	3	0.24
AUU (I)	360	1.85	ACU (T)	63	1.79	AAU (N)	191	1.83	AGU (S)	29	0.74
AUC (I)	30	0.15	ACC (T)	20	0.57	AAC (N)	18	0.17	AGC (S)	10	0.26
AUA (M)	256	1.81	ACA (T)	57	1.62	AAA (K)	82	1.61	AGA (S)	69	1.77
AUG (M)	27	0.19	ACG (T)	1	0.03	AAG (K)	20	0.39	AGG (S)	12	0.31
GUU (V)	78	2.05	GCU (A)	70	2.35	GAU (D)	68	1.74	GGU (G)	49	1.07
GUC (V)	10	0.26	GCC (A)	19	0.64	GAC (D)	10	0.26	GGC (G)	5	0.11
GUA (V)	59	1.55	GCA (A)	28	0.94	GAA (E)	63	1.43	GGA (G)	98	2.13
GUG (V)	5	0.13	GCG (A)	2	0.07	GAG (E)	25	0.57	GGG (G)	32	0.70

### 3.4 Transfer RNA and ribosomal RNA genes

Across the 37 species analyzed, total lengths ofthe 22 identified tRNAs fell between 1,405 and 1,439 bp. AT percentages spanned 78.08 to 81.12%, indicative of notable AT bias in these sequences. AT-skew values extended from −0.0088 to 0.0467; the newly sequenced species *N. s. sichuanyunnana* had an AT-skew of 0, indicating an equal number of adenine and thymine bases; GC-skew values ranged from 0.1370 to 0.1818.

The rRNA lengths of the 37 flea species ranged from 1,970 to 2,109 bp. The AT% ranged from 79.54 to 83.37%, showing a marked AT skew. The AT-skew ranged from −0.0236 to 0.0389,while the GC-skew ranged from 0.2986 to 0.3756. See Figure 3 and Table 2.

### 3.5 Analysis of phylogenetic trees

**Figure 7:**
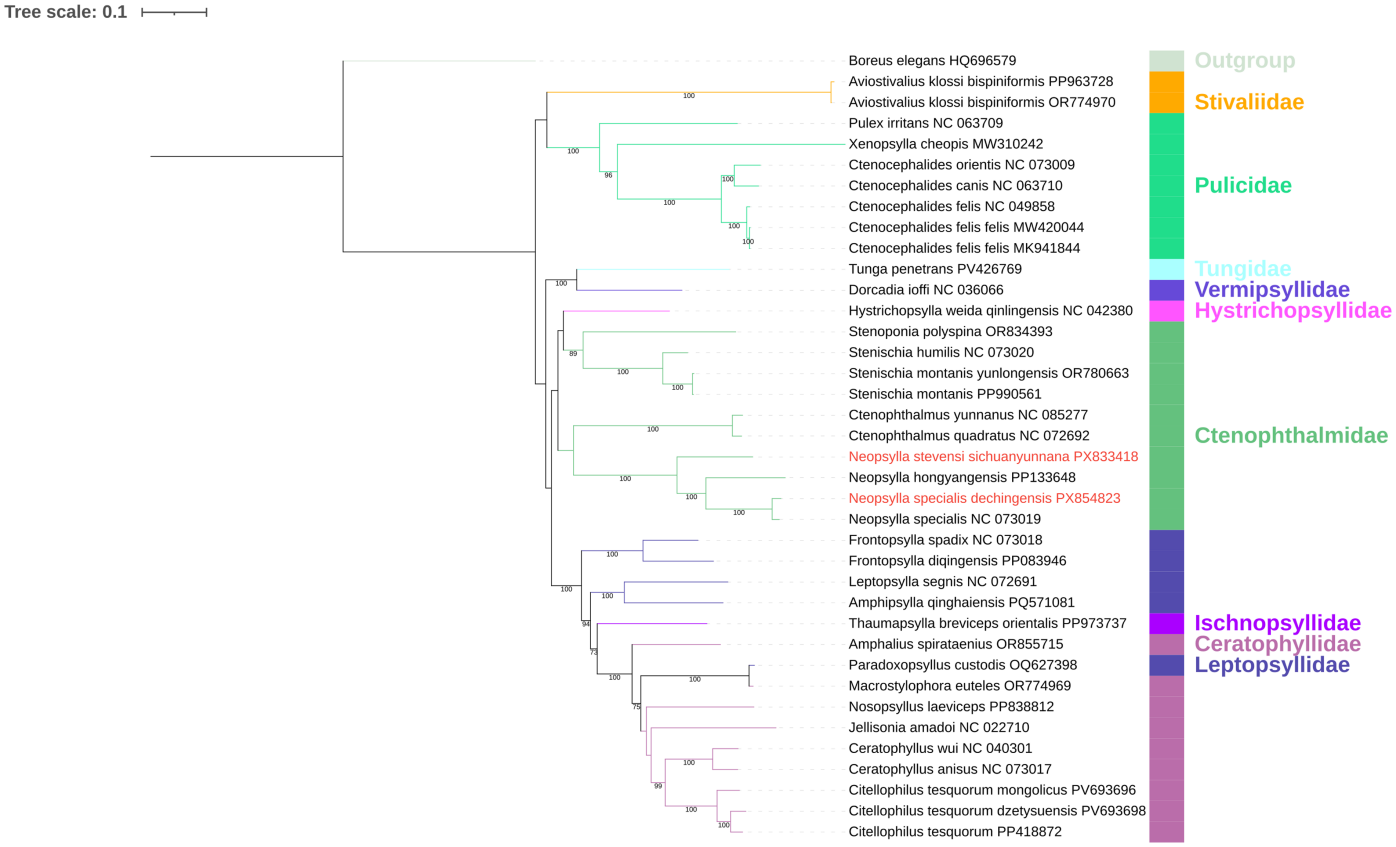
Maximum likelihood (ML) tree based on 13 PCGs from 37 flea species, with *Boreus elegans* (HQ696579) as the outgroup.

**Figure 8:**
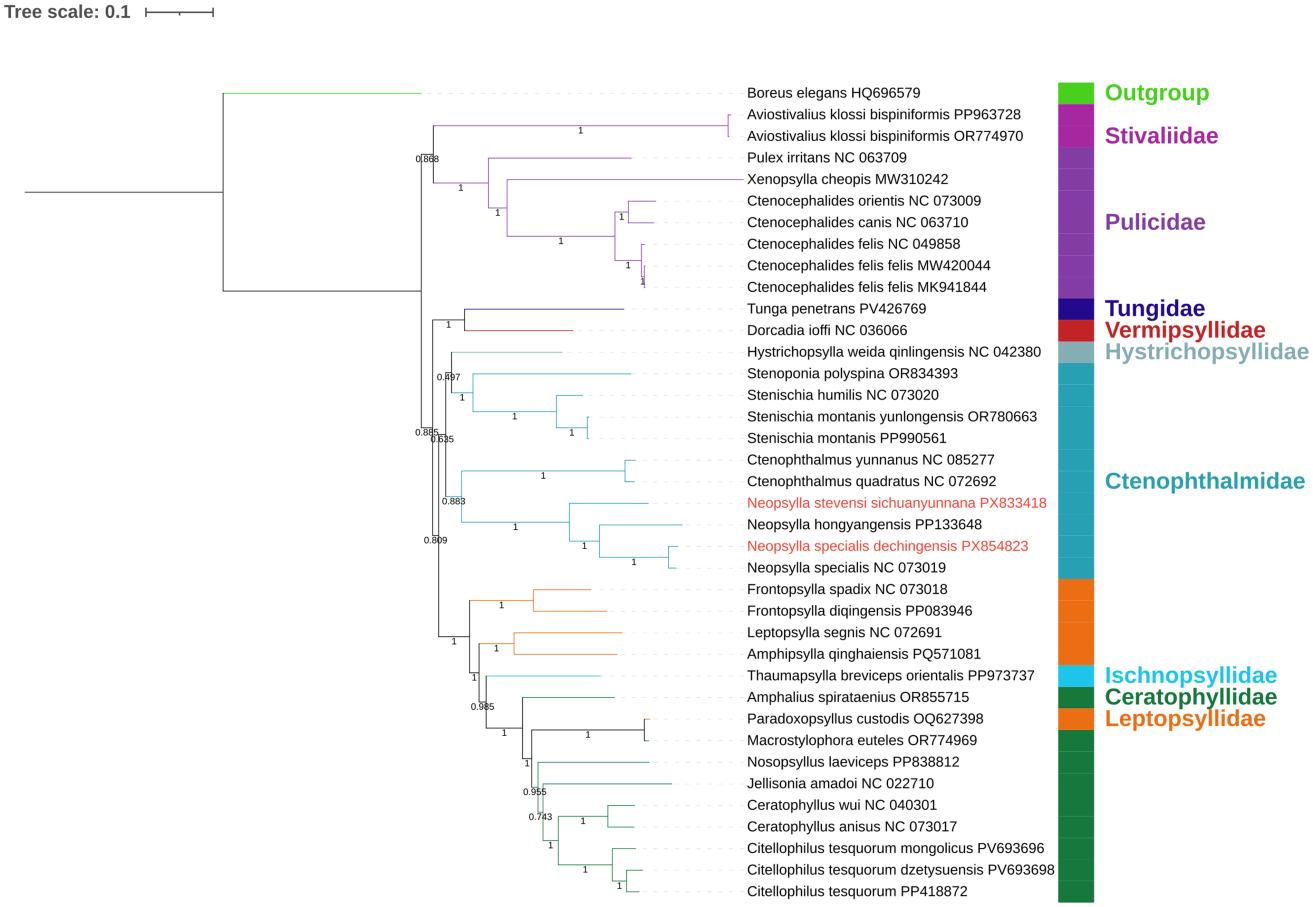
Bayesian inference (BI) based on 13 PCGs from 37 flea species, with *Boreus elegans* (HQ696579) as the outgroup.

The study constructed two phylogenetic trees based on newly sequenced data from two species, *N. s. dechingensis* and *N. s. sichuanyunnana*, along with 35 flea species from NCBI, using *B. elegans* (accession number: HQ696579) as the outgroup. Our findings demonstrated that the topological structures of the ML phylogeny inferred via IQ-TREE version 1.6.12 and the BI phylogeny generated with MrBayes v3.2.7 displayed extremely high congruence. The topology is mainly divided into two major clades: the first clade consists of Stivalidae and Pulicidae, while the second clade consists of Tungidae, Vermipsyllidae, Hystrichopsyllidae, Ctenophthalmidae, Ischnopsyllidae, Ceratophyllidae, and Leptopsyllidae. The second branch is further divided into two subclades: (Tungidae + Vermipsyllidae) and ((Hystrichopsyllidae + Ctenophthalmidae) + (Ischnopsyllidae + Ceratophyllidae + Leptopsyllidae)). Across both maximum likelihood (ML) and Bayesian inference (BI) phylogenetic assessments, the newly sequenced species *N. s. dechingensis* and *N. s. sichuanyunnana* were clustered within Ctenophthalmidae, and the self-expansion support values and posterior probabilities of each main branch were high, validating the reliability of the topological structure. The newly sequenced species *N. s. dechingensis* is most closely related to *Neopsylla specialis*, while *N. s. sichuanyunnana* and *Neopsylla hongyangensis* are sister groups. Both phylogenetic trees support the monophyly of Pulicidae, with clade support values and posterior probabilities of 100 and 1, respectively (see Figures 7 and 8).

## 4 Discussion

The shortage of sequenced mitochondrial genomes among flea taxa severely limits future efforts to prevent and control the diseases they cause. To combat this parasite, which poses a serious threat to human health, it is crucial to continuously expand the genome database to accurately identify and classify these species. The two newly sequenced species in this study, *N. s. dechingensis* (Accession No.: PX854823) and *N. s. sichuanyunnana* (accession number: PX833418), have been deposited in NCBI. Analysis of these two species revealed that their mitochondrial genomes contain 37 genes (22 tRNA, 13 PCGs, and 2 rRNA), consistent with the characteristics of other flea species (40). A systematic analysis incorporating the complete mitochondrial genomes of 35 other flea species from NCBI revealed that these 37 flea species exhibit significant AT skew, a finding consistent with most previous studies on fleas (41). Where the ratio between non-synonymous (Ka) and synonymous (Ks) substitution rates surpasses 1, it indicates that the corresponding coding gene undergoes positive selection, signifying the species possesses exceptional adaptability to environmental changes. If the ratio of Ka to Ks equals 1, it indicates neutral evolution of the protein-coding gene, with mutations not subject to selection. Conversely, when the Ka/Ks ratio falls below 1, this signifies that the corresponding coding gene is under negative selection, meaning that mutations detrimental to protein structure or function are eliminated by natural selection (42). The results of this study indicate that the Ka/Ks ratios for all 13 PCGs were less than 1, suggesting that they have undergone purifying selection, thereby maintaining the functional stability of proteins across 37 flea species. Both evolutionary rate and nucleotide diversity analyses indicate that the ATP8 gene is suitable for phylogenetic analysis among closely related species, while the COX1 gene, due to its high sequence conservation, can serve as a DNA barcode marker for species identification (43, 44). Dysfunction of ATP8 disrupts the assembly and activity of ATP synthase, thereby impairing oxidative phosphorylation (OXPHOS)—the primary pathway by which cells generate ATP. Consequently, ATP8 is not only a structural protein but also a key functional link between the mitochondrial genome and cellular energy metabolism (45). ATN-type initiation codons represent the predominant form across the class Insecta; the start codons of *N. s. dechingensis* and *N. s. sichuanyunnana* conform to this pattern, with only the latter’s ND4 gene employing the non-standard start codon GTG. Incomplete termination codons ending in TA or T are fully corrected via post-transcriptional polyadenylation, which generates a complete functional TAA stop codon (46).

The results of two phylogenetic trees constructed using 37 flea species and 13 PCGs from *B. elegans* as an outgroup are consistent with previous studies that used five molecular genetic markers to conduct phylogenetic analyses, which generally support the monophyly of Pulicidae and the paraphyly of Ctenophthalmidae. However, this differs from the results of Zhou et al. (47), who classified Leptopsyllidae and Ceratophyllidae as monophyletic groups. It is worth noting that in recent years, some studies have identified the Ctenophthalmidae as monophyletic using PCGRNA-ML (codon split) phylogenetic trees (48). In this study, the Leptopsyllidae are paraphyletic, which is consistent with the findings of most studies indicating that they are paraphyletic (49–51). Controversy surrounding the Hystrichopsyllidae has long been intense in the study of fleas, with some classifying the family as monophyletic (52), while others, consistent with the findings of this study, classify it as paraphyletic (3, 53). However, since this study includes only one sequence from the Hystrichopsyllidae family, it cannot definitively confirm that it is a paraphyletic group.

Although there is a great diversity of flea species, very few have complete mitochondrial genomes. Compared to partial mitochondrial genomes, complete mitochondrial genomes offer greater sensitivity and discriminatory power when studying evolutionary relationships among closely related species (54). This research addresses the existing gap in the complete mitochondrial genome data for *N. s. dechingensis* and *N. s. sichuanyunnana*, a finding that holds substantial importance for the precise identification of flea species and the future prevention and control of disease transmission. A limitation of this study is the small sample size, which resulted in an incomplete analysis; therefore, future research should collect as many samples as possible to conduct more in-depth analyses and address this shortcoming.

## 5 Conclusion

This study covers the entire workflow of assembly, annotation, and sequencing, providing data support for the taxonomic classification of flea species. It analyzed the mitochondrial genomic characteristics and phylogenetic relationships of *N. s. dechingensis* and *N. s. sichuanyunnana*, expanded the mitochondrial genome database for fleas, and laid the foundation for developing more effective disease prevention measures against flea-borne diseases in future research.

## Data Availability

The datasets presented in this study can be found in online repositories. The names of the repository/repositories and accession number(s) can be found below: https://www.ncbi.nlm.nih.gov/, PX833418 https://www.ncbi.nlm.nih.gov/, PX854823.

## Footnotes

1
https://github.com/OpenGene/fastp

2
https://github.com/Kinggerm/GetOrganelle

3
https://github.com/rrwick/Bandage

4
http://mitos.bioinf.uni-leipzig.de/

## Supplementary material

The Supplementary material can be found online at: https://www.frontiersin.org/articles/10.3389/fvets.2026.1865746/full#supplementary-material
