# Supplementary material for: The first complete mitochondrial genome sequences of Neopsylla specialis dechingensis and Neopsylla stevensi sichuanyunnana, with an assessment of their phylogenetic placement
Source: Front Vet Sci. 2026 Jun 23;13:1865746. doi: 10.3389/fvets.2026.1865746 (PMC13339032; doi:10.3389/fvets.2026.1865746)
Supplement: Supplementary file 1 [file Table_1.DOCX]

Supplementary Material

## Supplementary Table 1

| **Feature** | **Strand** | **Position(start-end)** | **Length(bp)** | **Initiation_codon** | **Stop_codon** | **Anticodon** |
| --- | --- | --- | --- | --- | --- | --- |
| trnI | + | 1-63 | 63 |  |  | GAT |
| trnQ | - | 108-176 | 69 |  |  | TTG |
| trnM | + | 231-295 | 65 |  |  | CAT |
| nad2 | + | 296-1,306 | 1,011 | ATT | TAA |  |
| trnW | + | 1,305-1,368 | 64 |  |  | TCA |
| trnC | - | 1,361-1,421 | 61 |  |  | GCA |
| trnY | - | 1,422-1,484 | 63 |  |  | GTA |
| cox1 | + | 1,482-3,017 | 1,536 | ATC | TAA |  |
| trnL2 | + | 3,022-3,084 | 63 |  |  | TAA |
| cox2 | + | 3,086-3,766 | 681 | ATG | TAG |  |
| trnK | + | 3,769-3,838 | 70 |  |  | CTT |
| trnD | + | 3,838-3,900 | 63 |  |  | GTC |
| atp8 | + | 3,901-4,068 | 168 | ATT | TAA |  |
| atp6 | + | 4,062-4,733 | 672 | ATG | TAA |  |
| cox3 | + | 4,733-5,515 | 783 | ATG | TAA |  |
| trnG | + | 5,516-5,577 | 62 |  |  | TCC |
| nad3 | + | 5,578-5,928 | 351 | ATT | TAG |  |
| trnA | + | 5,927-5,989 | 63 |  |  | TGC |
| trnR | + | 5,990-6,052 | 63 |  |  | TCG |
| trnN | + | 6,053-6,116 | 64 |  |  | GTT |
| trnS1 | + | 6,117-6,185 | 69 |  |  | TCT |
| trnE | + | 6,186-6,249 | 64 |  |  | TTC |
| trnF | - | 6,248-6,311 | 64 |  |  | GAA |
| nad5 | - | 6,313-8,028 | 1,716 | ATG | TTA |  |
| trnH | - | 8,030-8,091 | 62 |  |  | GTG |
| nad4 | - | 8,092-9,430 | 1,339 | ATG | T |  |
| nad4l | - | 9,424-9,717 | 294 | ATG | TAA |  |
| trnT | + | 9,720-9,784 | 65 |  |  | TGT |
| trnP | - | 9,785-9,847 | 63 |  |  | TGG |
| nad6 | + | 9,856-10,365 | 510 | ATT | TAA |  |
| cob | + | 10,365-11,498 | 1,134 | ATG | TAA |  |
| trnS2 | + | 11,500-11,564 | 65 |  |  | TGA |
| nad1 | - | 11,583-12,524 | 942 | ATG | TAA |  |
| trnL1 | - | 12,526-12,587 | 62 |  |  | TAG |
| rrnL | - | 12,588-13,851 | 1,264 |  |  |  |
| trnV | - | 13,885-13,950 | 66 |  |  | TAC |
| rrnS | - | 13,951-14,740 | 790 |  |  |  |
| OH | + | 16,455-17,144 | 690 |  |  |  |

**Supplementary Table 1A:**Basic mitochondrial information of *Neopsylla specialis dechingensis.*

| **Feature** | **Strand** | **Position**  **(start-end)** | **Length(bp)** | **Initiation_codon** | **Stop_codon** | **Anticodon** |
| --- | --- | --- | --- | --- | --- | --- |
| trnI | + | 486-548 | 63 |  |  | GAT |
| trnQ | - | 572-640 | 69 |  |  | TTG |
| trnM | + | 722-786 | 65 |  |  | CAT |
| nad2 | + | 787-1,797 | 1,011 | ATC | TAA |  |
| trnW | + | 1,796-1,859 | 64 |  |  | TCA |
| trnC | - | 1,852-1,912 | 61 |  |  | GCA |
| trnY | - | 1,913-1,974 | 62 |  |  | GTA |
| cox1 | + | 1,972-3,507 | 1,536 | ATC | TAA |  |
| trnL2 | + | 3,512-3,575 | 64 |  |  | TAA |
| cox2 | + | 3,576-4,256 | 681 | ATG | TAA |  |
| trnK | + | 4,259-4,328 | 70 |  |  | CTT |
| trnD | + | 4,328-4,394 | 67 |  |  | GTC |
| atp8 | + | 4,395-4,562 | 168 | ATC | TAA |  |
| atp6 | + | 4,556-5,227 | 672 | ATG | TAA |  |
| cox3 | + | 5,227-6,009 | 783 | ATG | TAA |  |
| trnG | + | 6,010-6,071 | 62 |  |  | TCC |
| nad3 | + | 6,069-6,422 | 354 | ATA | TAG |  |
| trnA | + | 6,421-6,483 | 63 |  |  | TGC |
| trnR | + | 6,483-6,544 | 62 |  |  | TCG |
| trnN | + | 6,545-6,608 | 64 |  |  | GTT |
| trnS1 | + | 6,609-6,676 | 68 |  |  | TCT |
| trnE | + | 6,677-6,740 | 64 |  |  | TTC |
| trnF | - | 6,739-6,803 | 65 |  |  | GAA |
| nad5 | - | 6,803-8,520 | 1,718 | ATG | TA |  |
| trnH | - | 8,522-8,583 | 62 |  |  | GTG |
| nad4 | - | 8,584-9,922 | 1,339 | GTG | T |  |
| nad4l | - | 9,916-10,206 | 291 | ATG | TAA |  |
| trnT | + | 10,209-10,273 | 65 |  |  | TGT |
| trnP | - | 10,274-10,336 | 63 |  |  | TGG |
| nad6 | + | 10,339-10,851 | 513 | ATT | TAA |  |
| cob | + | 10,851-11,984 | 1,134 | ATG | TAA |  |
| trnS2 | + | 11,986-12,050 | 65 |  |  | TGA |
| nad1 | - | 12,069-13,013 | 945 | ATG | TAA |  |
| trnL1 | - | 13,015-13,076 | 62 |  |  | TAG |
| rrnL | - | 13,077-14,338 | 1,262 |  |  |  |
| trnV | - | 14,372-14,438 | 67 |  |  | TAC |
| rrnS | - | 14,439-15,232 | 794 |  |  |  |
| OH | + | 15,297-15,357 | 61 |  |  |  |

**Supplementary Table 1B:**Basic mitochondrial information of *Neopsylla stevensi sichuanyunnana.*
